# Phospholipase D functional ablation has a protective effect in an Alzheimer’s disease *Caenorhabditis elegans* model

**DOI:** 10.1038/s41598-018-21918-5

**Published:** 2018-02-23

**Authors:** Francisca Vaz Bravo, Jorge Da Silva, Robin Barry Chan, Gilbert Di Paolo, Andreia Teixeira-Castro, Tiago Gil Oliveira

**Affiliations:** 1 Life and Health Sciences Research Institute (ICVS), School of Medicine, University of Minho, Braga, Portugal; 2 ICVS/3B’s—PT Government Associate Laboratory, Braga/Guimarães, Portugal; 3 Department of Pathology and Cell Biology, Taub Institute for Research on Alzheimer’s Disease and the Aging Brain, Columbia University Medical Center, New York, New York, 10032 USA; 4 Present Address: Denali Therapeutics Inc., South San Francisco, CA 94080 USA

## Abstract

Phospholipase D (PLD) is a key player in the modulation of multiple aspects of cell physiology and has been proposed as a therapeutic target for Alzheimer’s disease (AD). Here, we characterize a PLD mutant, *pld-1*, using the *Caenorhabditis elegans* animal model. We show that *pld-1* animals present decreased phosphatidic acid levels, that PLD is the only source of total PLD activity and that *pld-1* animals are more sensitive to the acute effects of ethanol. We further show that PLD is not essential for survival or for the normal performance in a battery of behavioral tests. Interestingly, *pld-1* animals present both increased size and lipid stores levels. While ablation of PLD has no important effect in worm behavior, its ablation in an AD-like model that overexpresses amyloid-beta (Aβ), markedly improves various phenotypes such as motor tasks, prevents susceptibility to a proconvulsivant drug, has a protective effect upon serotonin treatment and reverts the biometric changes in the Aβ animals, leading to the normalization of the worm body size. Overall, this work proposes the *C*. *elegans* model as a relevant tool to study the functions of PLD and further supports the notion that PLD has a significant role in neurodegeneration.

## Introduction

Alzheimer’s disease (AD) is the most common form of late-onset dementia. One of the main pathological hallmarks of AD is the accumulation of amyloid-beta (Aβ) plaques in the brain, derived from the sequential cleavage of the amyloid precursor protein (APP) by beta and gamma secretases^1^. Presently, there are no effective therapeutical options for AD and one potential strategy being pursued is to block Aβ pathological signaling. Remarkably, using amyloidogenesis AD mouse models, it was shown that the genetic ablation of a myriad of putative Aβ signaling downstream players, such as tau^2^, PrP^3^, GIVA-phospholipase A2 (GIVA-PLA2)^4^ or phospholipase D2 (PLD2)^5^ ameliorates rodent behavioral cognitive deficits, independently of APP processing or Aβ levels modulation.

Lipids are a major constituent of the brain and specifically signaling lipids have been shown to regulate brain functioning and to modulate various neurodegenerative processes^6^. Indeed, Aβ has been shown to activate a group of lipid modulating enzymes, such as PLC^7^, GIVA-PLA2^4^ and PLD^8^. While the PLC and PLA2 pathways are well studied, less is known about the PLD pathway. In mammals, six members of the PLD superfamily have been identified^9^. From these, there are two canonical PLD isoenzymes, PLD1 and PLD2, which are structurally similar and enzymatically both convert phosphatidylcholine (PC) to phosphatidic acid (PA), but differ in their intracellular localization and mechanisms of regulation^8,10^. Interestingly, in the presence of primary alcohols, such as ethanol, PLD preferentially uses it as a substrate, producing a specific lipid, phosphatidylethanol (PEtOH), which is often used to measure PLD activity^8^. Even though PLD2 has been shown to be involved in Aβ signaling^5^, PLD1 has been proposed to modulate APP trafficking and processing^11,12^. As an approach to understand the role of the PLD pathway in physiology and in a pathological context, the study of PLD mutant associated phenotypes in several model organisms, such as nematodes, drosophila^13,14^ and mice^5,15^, can give key insights. Importantly, while in mice there are two PLD isoenzymes, in drosophila and in nematodes there is only one PLD enzyme^16^.

The study of neurodegenerative diseases in simple organisms, such as *Caenorhabditis elegans*, when appropriately adapted to the nematode physiology, provides a powerful tool in the identification of relevant pathological pathways^17^. For instance, the strain CL2355, which overexpresses human Aβ in neurons and presents multiple aberrant behaviors^18–20^, has been proposed to be an effective model to study Aβ pathological signaling.

Here, we studied the impact of PLD genetic ablation in *C*. *elegans* in a physiological context and upon crossing it with an AD-like model. We showed that PLD ablation leads to a decrease in PA levels and that PLD is the only source of PEtOH upon ethanol treatment. While we found no major behavioral deficits, we observed a small increase in the worm volume. Remarkably, PLD ablation restored not only worm volume in an AD-like model, but also had a protective effect in motor behaviors and in sensitivity to serotonin and pharmacologically-induced seizures, suggesting a disease-modifying role for PLD in *C*. *elegans*.

## Results

### *pld-1* worms present decreased PA levels and PLD activity

**Figure 1:**
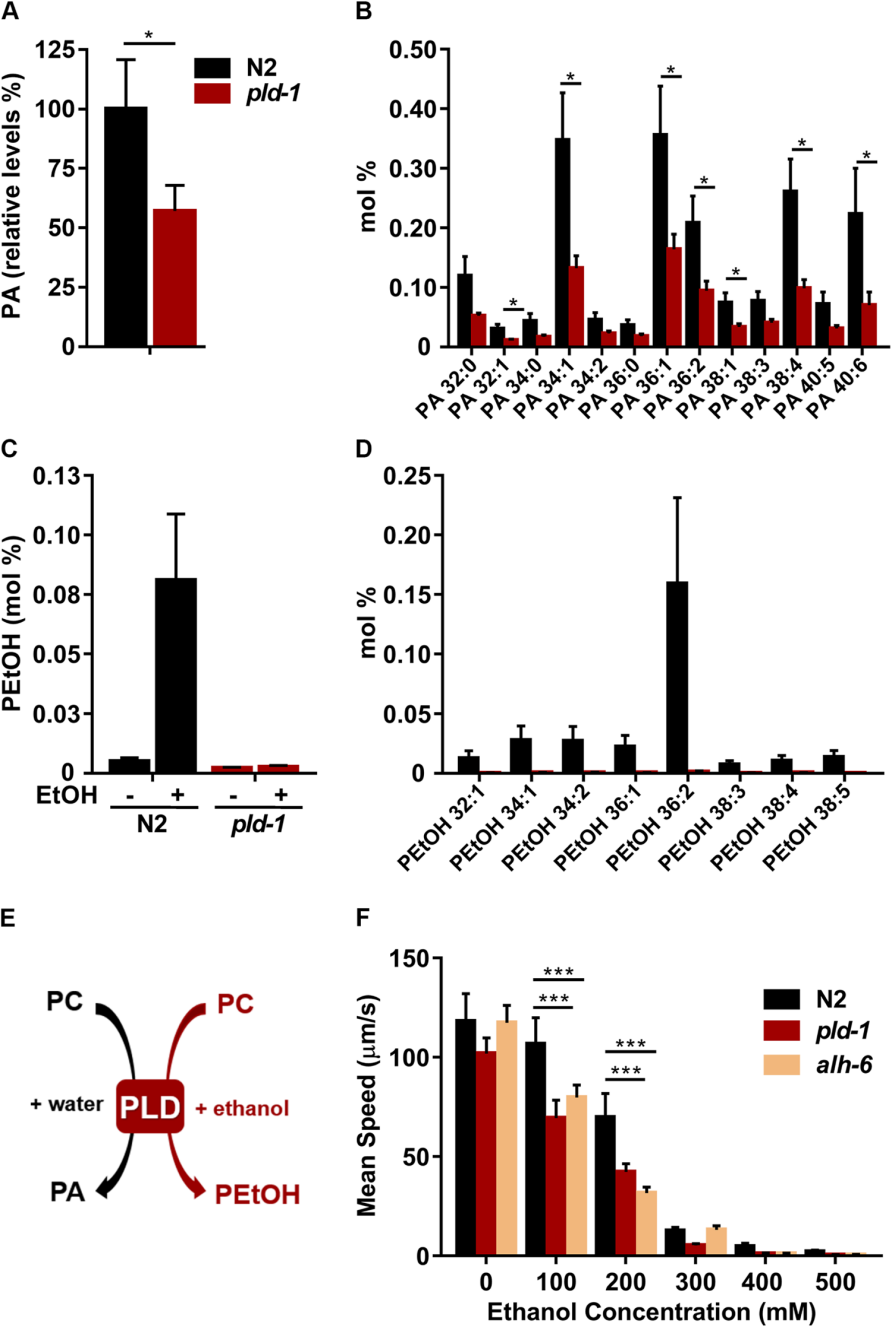
*pld-1* animals show decreased PA levels and PLD activity. (**A**) *pld-1* ablation causes a 50% reduction on total phosphatidic acid (PA) relative levels in *C*. *elegans*. (**B**) Relative amounts of the different PA species measured in *pld-1* and WT animals. Levels of PA were quantified by Liquid Chromatography Mass Spectrometry (LC-MS) analysis (n = 6). (**C**) Relative amounts of total PEtOH (**D**) and of the different PEtOH species measured in *pld-1* and WT animals (n = 6) and quantified by LC-MS. (**E**) Graphical representation of the synthesis reaction of phosphatidylethanol (PEtOH) *in vivo*. PLD has an increased affinity to primary alcohols, leading to the generation of PA in the presence of water, or PEtOH if in the presence of ethanol. This reaction is the only source of PEtOH and can be used as a measure of PLD activity. (**F**) *pld-1* animals are more susceptible than WT to the acute effects of ethanol (at 100 and 200 mM), similarly to the ethanol-susceptible strain *alh-6*, as shown by the decrease in the mean speed of the animals (n = 6). Values denote mean ± SEM (*p < 0.05, ***p < 0.001). The nomenclature for PA and PEtOH fatty acid species composition is expressed as total chain length: number of unsaturated bonds.

In order to address the role of PLD in *C*. *elegans* lipid metabolism (Supplementary Fig. S1), we performed a lipidomic analysis to biochemically characterize the *pld-1 C*. *elegans* model. Since PLD converts PC to PA, we measured the levels of PA in N2 and *pld-1* worms, using liquid chromatography-mass spectrometry (LC-MS). We found that PLD mutants present a ~50% decrease in total PA levels (Fig. 1A). Specifically, six molecular species of PA (based on their different fatty acyl composition), namely PA 32:1, PA 34:1, PA 36:1, PA 38:1, PA 38:4 and PA 40:6 were diminished, while a trend for a decrease was observed for the other species (Fig. 1B). Since PLD is not the only source of PA, we developed an *in vivo* PLD activity assay, relying on the incubation of worms for 1 hour with 1% ethanol and subsequent measurement of phosphatidylethanol (PEtOH), a lipid uniquely produced by PLD. We found a major decrease in multiple PEtOH species in *pld-1* comparing with N2 worms (Fig. 1D), which indicates that PLD is the only source of PEtOH in *C*. *elegans*. Next, we performed an ethanol susceptibility assay, since PLD uses ethanol as a substrate to produce PEtOH. Animals were exposed to different doses of ethanol and their mean speed was evaluated. An aldehyde dehydrogenase mutant (*alh-6*) was used as an ethanol-sensitive control strain and we observed that in two doses (100 and 200 mM), *pld-1* worms were more susceptible than N2 animals to the acute ethanol effects, similarly to *alh-6* animals (Fig. 1F). Moreover, in order to test if the fraction of ethanol metabolized by PLD would be significant in the context of total levels of ethanol, we compared total ethanol levels in N2 animals and with *pld-1* animals after one hour of treatment and we observed no significant differences (1.00 ± 0.10 and 0.98 ± 0.08, in N2 vs. *pld-1*, resp. as relative levels to control, from three independent experiments n = 250 animals per experiment). Taken together, these results show that PLD ablation in worms leads to decreased PA levels, decreased total PLD activity and impacts the sensitivity to ethanol acute effects.

### PLD ablation causes no gross phenotypes in *C*. *elegans*

**Figure 2:**
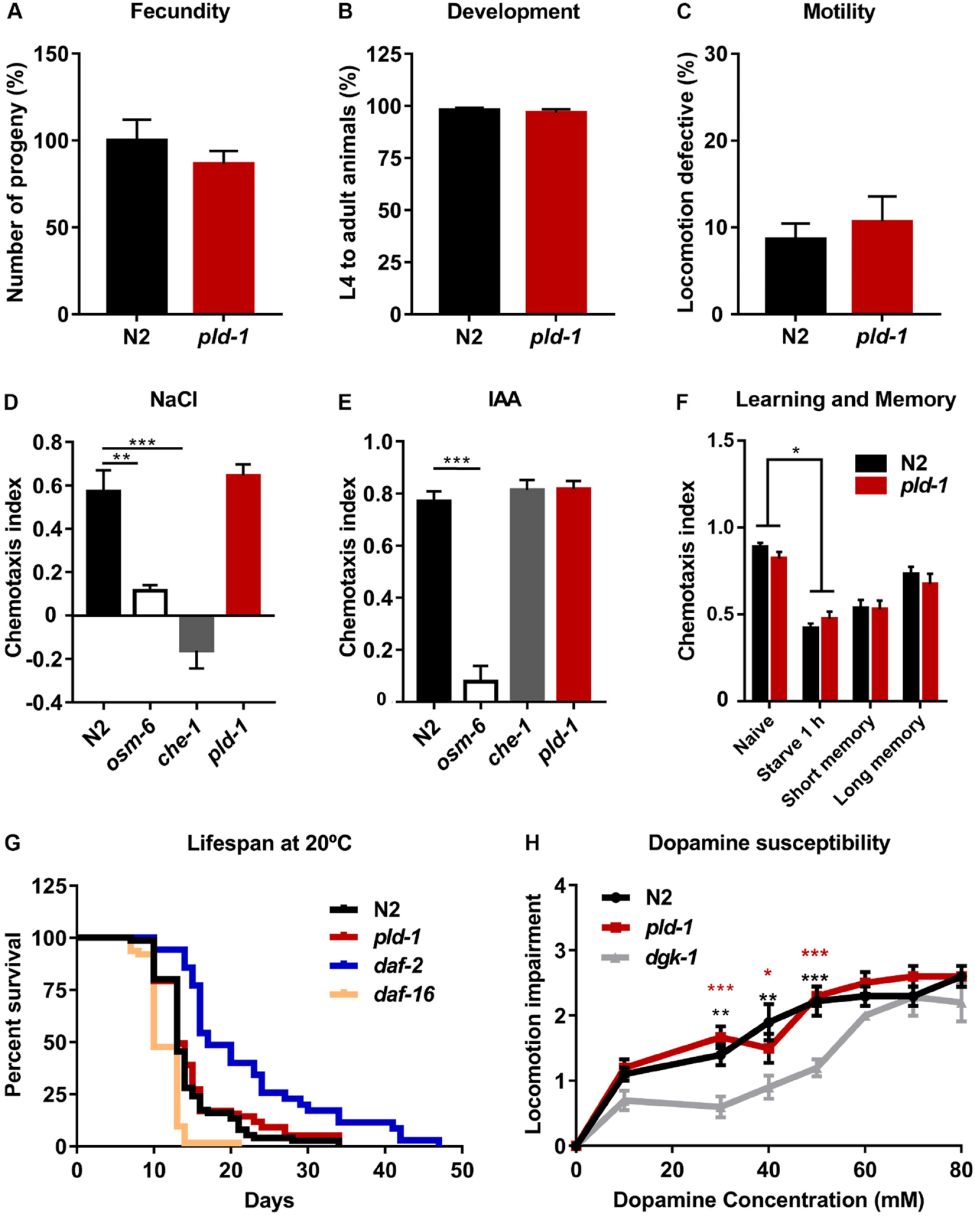
*pld-1* animals show no major phenotypic alterations. (**A**–**C**) Effect of PLD ablation on *C*. *elegans* egg-laying, development and locomotion behavior. (**A**) Number of progeny of adult hermaphrodite N2 and *pld-1* animals for 5 h. The progeny of 10 worms was counted per experiment (n = 3). (**B**) Percentage of L4 to adult N2 and *pld-1* animals after 48 h post egg laying. At least 150 animals were averaged per dataset (n = 3). (**C**) The percentage of locomotion defective age-synchronized adult animals (day 3 post-hatching) was measured by scoring the animals that remain inside a 10 mm circle, 1 min after being placed on its center. The number of animals used per trial was 10 (n = 3). (**D**,**E**) *pld-1* worms have a normal chemotaxis response. Chemotaxis response to NaCl and isoamyl alcohol (IAA) was evaluated in N2, *pld-1*, *che-1* and *osm-6* animals. Mutant strains unable to detect NaCl (*osm-6* and *che-1*) and IAA (*osm-6*) were used as negative controls. Three independent experiments were performed (n = 200 worms per assay). (**F**) *pld-1* animals have no deficit in an associative learning task. N2 and *pld-1* short-term and long-term associative memory profile after 1 h and 24 h of conditioning. Representative example of three independent experiments is shown (n = approximately 100 worms per chemotaxis assay plate). (**G**) *pld-1* worms have a normal lifespan. Kaplan-Meier survival curve of *pld-1* worms, show no difference in the median lifespan. Survival rate was scored everyday and is expressed as percentage of survival. *daf-2* and *daf-16* strains were used as long and short-lived controls, respectively. The data results from the analysis of 100 worms per strain in 3 independent experiments. (**H**) Dose-response curves measuring paralysis induced by exogenous dopamine. Locomotion impairment of animals moving 30 min after being placed on agar plates containing the indicated concentrations of dopamine is shown. N2 and *pld-1 animals* show no differences to N2 in sensitivity to dopamine, whereas *dgk-1* animals exhibit resistance to paralysis induced by exogenous dopamine. The data for three independent experiments is represented (n = 10 animals for each dopamine concentration). Values denote means ± SEM, ns-non-significant. (**p ≤ 0.01, ***p ≤ 0.001).

In order to perform a characterization of *pld-1* animals, we ran a battery of tests. We first found that the number of progeny (Fig. 2A) and subsequent development (Fig. 2B), assessed by the percentage of adult worms in the total population, was unchanged. Additionally, motor behavior, which was evaluated by assessing locomotion defects, was not affected in *pld-1* worms (Fig. 2C). Considering that *C*. *elegans* has different types of sensory neurons, we performed chemotaxis assays to evaluate the ability of *pld-1* worms to approach or avoid a compound, and we observed no differences in the chemotactic responses for all tested chemicals (Fig. 2D and E). Moreover, we performed an associative learning task and no deficits were observed in *pld-1* worms (Fig. 2F). In order to evaluate the effect of PLD ablation on survival, we performed a lifespan assay. No differences were observed in the median lifespan of *pld-1* worms when compared to N2 (Fig. 2G) (Supplementary Table S2). Furthermore, in order to explore the role of PLD in PA-mediated cell signaling, we performed a dopamine susceptibility assay. Dopamine binds to dopaminergic receptors coupled to a G-protein, activating several effectors, including PLC. The activation of PLC leads to an increase in diacylglycerol (DAG) that could then be converted to PA. As previously described, DAG kinase 1 mutant worms (*dgk-1*) were shown to be resistant to dopamine^21^. However, even though DGK and PLD are both sources of PA, *pld-1* animals presented no differences in the susceptibility to dopamine induced locomotion impairment (Fig. 2H). Thus, our observations suggest that PLD ablation has no impact in worm lifespan and causes no gross behavioral alterations.

### PLD modulates the body size of *C*. *elegans*

**Figure 3:**
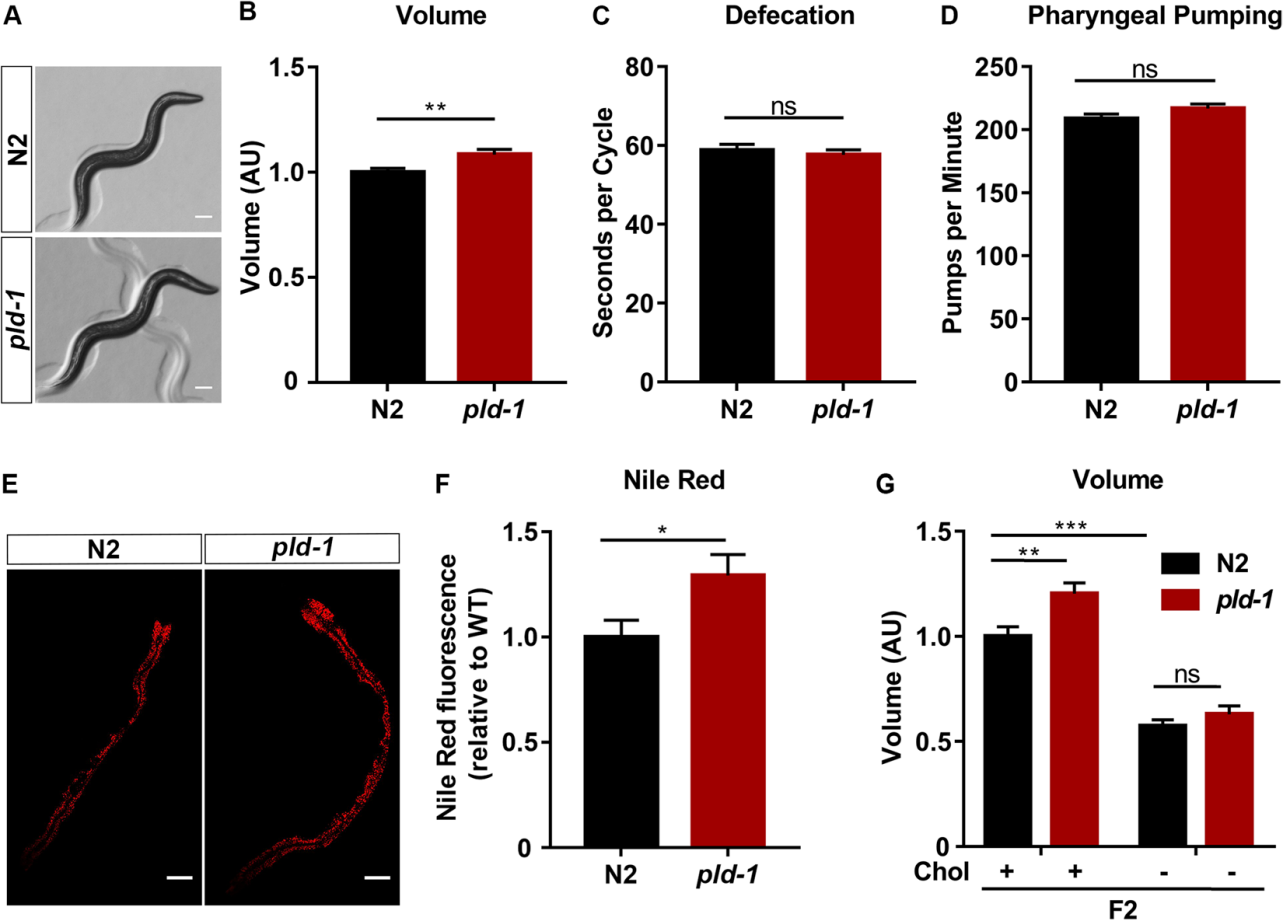
PLD modulates *C*. *elegans* volume. (**A**,**B**) *pld-1* worms have an increase in body volume. Representative photos (**A**) and graphic analysis of volume (**B**) of N2 and *pld-1* animals at day 3 after egg laying. The scale bar represents 100 µm. (**B**) 40 animals were analyzed per strain in 3 independent experiments. (**C**,**D**) *pld-1* worms have no alterations in physiological parameters, namely (**C**) defecation cycle length and (**D**) pharyngeal pumping rate (n = 3, 10 animals per assay). (**E**,**F**) *pld-1* worms have changes in lipid balance. Nile Red staining representative photos (**E**) and quantification of Nile Red fluorescence of N2 and *pld-1* worms at day three post-hatching. Photographs were taken at 60 × magnification. Scale bar represents 100 µm. (**G**) *pld-1* and N2 animals present similar volumes upon cholesterol depletion. Volume analysis of F2 generation (N2 and *pld-1*) animals at 72 h after egg laying, in the presence or absence of cholesterol. The data results from the analysis of 45 worms per strain in 4 independent experiments. Values denote means ± SEM. (*p ≤ 0.05, **p ≤ 0.01, ***p ≤ 0.001).

As part of our characterization of PLD mutants, we performed a biometric analysis and interestingly we observed that *pld-1* animals consistently presented an increase (~10%) volume of the body when compared to N2 worms (Fig. 3A and B). Importantly, we saw no differences in the defecation cycles (Fig. 3C) and the pharyngeal pumping rates (Fig. 3D) of well-fed mutants. Using Nile red fluorescence, an indicator of neutral lipid content, we showed that *pld-1* animals presented an increase (~30%) in lipid accumulation (Fig. 3E and F). It was previously shown that cholesterol deprivation leads to developmentally-induced volume reduction in the F2 generation^22,23^. We evaluated the impact of PLD ablation in this lipid induced developmental volume deficit and we observed that *pld-1* animals had no volume differences compared to N2 in cholesterol-deprived F2 generation animals (Fig. 3G). These results suggest that PLD regulates worm body size and lipid stores.

### PLD functional ablation ameliorates Aβ phenotypes in an AD-like model

**Figure 4:**
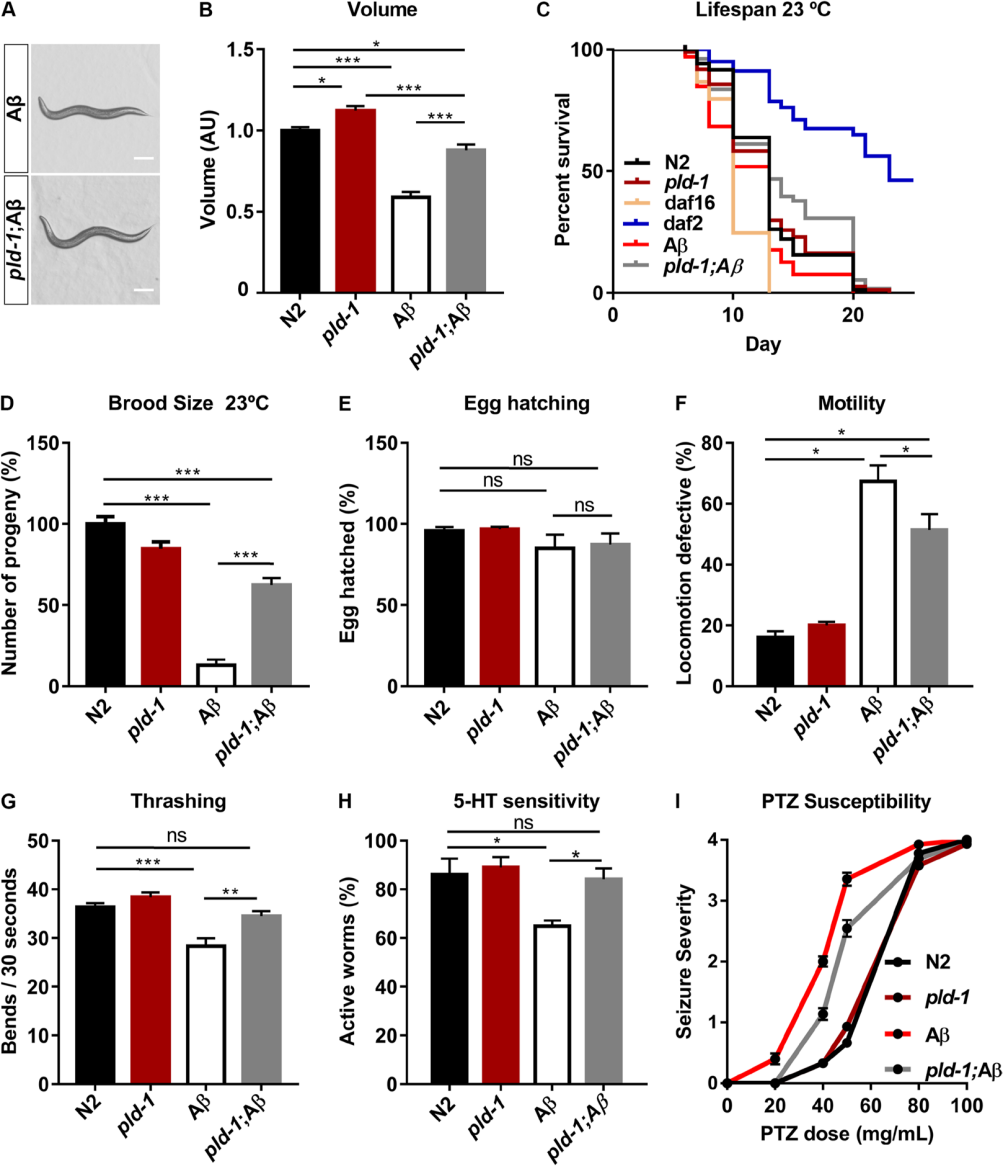
PLD functional ablation ameliorates Aβ induced phenotypes. (**A**) Representative photos of Aβ and *pld-1*;Aβ and volume (**B**) of N2, *pld-1*, Aβ and *pld-1*;Aβ animals at day 3 after egg laying. The scale bar represents 100 µm. (**B**) Aβ transgenic worms have a decrease in body size, which is partially renormalized upon PLD ablation. The data results from the analysis of 45 worms per strain in 4 independent experiments. Values denote means ± SEM. (*p ≤ 0.05, **p ≤ 0.01, ***p ≤ 0.001). (**C**) Effects of neuronal Aβ expression on adult lifespan at 23 °C. The experiment was conducted twice with 100 worms per strain. Representative example of two independent experiments is shown. Kaplan-Meier survival curve of Aβ and *pld-1*; Aβ worms, showing differences in the median lifespan compared with N2 and *pld-1* animals. The mean adult lifespan of *pld-1*; Aβ animals was significantly longer than Aβ transgenic animals. (**D**–**I**) Neuronal expression of Aβ in *C*. *elegans* leads to a defect in brood size(**D**), locomotion (**F**), decrease in thrashes over time (**G**), serotonin sensitivity (**H**) and pentylenetetrazol (PTZ) susceptibility (**I**), when compared to the N2 and *pld-1* animals. (**D**) Improvement in brood size due to ablation of PLD in Aβ animals. Total number of progeny of adult hermaphrodite N2, *pld-1*, Aβ and *pld-1*; Aβ worms for 8 days. The progeny of 15 worms was counted per experiment (n = 2). (**E**) Egg viability was not significantly affected by neuronal expression of Aβ (n = 3). (**F**) Aβ worms have a motility defect partially recovered by PLD ablation. Percentage of uncoordinated N2, *pld-1*, Aβ and *pld-1*; Aβ adult hermaphrodite animals was measured by scoring the animals that remain inside 10 mm circle after 1 min (n = 4 experiments, 10 worms per strain and experiment). (**G**) The number of body thrashes per 30 seconds was partially rescued by PLD ablation in Aβ transgenic animals (n = 4 experiments, 10 worms per strain and experiment). (**H**) PLD ablation in Aβ animals leads to a decrease in hypersensitivity to 1 mM serotonin caused by Aβ (n = 4 experiments, 60 worms per strain and experiment). (**I**) PLD ablation reduces seizure-severity induced by PTZ in Aβ transgenic worms. PTZ susceptibility of worms were scored after exposure to the concentrations of 20, 40, 50, 80 and 100 mg/mL of PTZ (n = 4 experiments, 15 animals per dose/strain/experiment). Values denote means ± SEM. (*p ≤ 0.05, **p ≤ 0.01, ***p ≤ 0.001).

We had previously observed that the CL2355 strain (Aβ), which has a pan-neuronal expression of Aβ after induction by temperature up-shift to 23 °C, presented a major decrease (~40%) in worm volume (Fig. 4A). We then crossed the *pld-1* strain with the Aβ strain to test the impact of PLD functional ablation in an AD-like model. Remarkably, *pld-1*; Aβ animals presented significantly higher volumes than Aβ worms (Fig. 4B). Taking into account our previous work showing that *Pld2* genetic ablation had a protective effect in synaptic and behavioral deficits in an AD amyloidogenesis mouse model^5^, we tested if the functional ablation of PLD in worms had not only an effect in the volume of Aβ worms but also in other phenotypes. First, we performed a lifespan assay, conducted at 23 °C, in order to evaluate the effect of neuronal Aβ expression on overall survival. We observed a significant decrease in the median life span of Aβ worms compared to N2 and interestingly *pld-1*; Aβ animals presented increased median survival relative to Aβ worms (Fig. 4C) (Supplementary Table S3). Moreover, while we observed diminished egg-laying in Aβ animals (Fig. 4D), the ablation of PLD in the Aβ background resulted in an increase in the number of laid eggs (Fig. 4D) (Supplementary Fig. S3). Egg viability was not affected by either Aβ or PLD ablation (Fig. 4E). Concerning motor task assessment, both crawling and swimming were shown to be impaired in Aβ worms and, again, PLD ablation partially restored these deficits in *pld-1*; Aβ worms (Fig. 4F and G). We also evaluated the sensitivity of animals to serotonin, since it was previously shown that Aβ transgenic worms are more sensitive to this neurotransmitter^18^. Notably, *pld-1*; Aβ worms presented reduced sensitivity to serotonin-induced impairments when comparing with Aβ worms (Fig. 4H). Finally, we performed a pharmacologically-induced pro-excitatory assay using pentylenetetrazol (PTZ), a GABA receptor antagonist that increases neuronal excitability by disrupting the normal excitatory/inhibitory balance^24^. We exposed worms to different doses of PTZ and measured seizure severity. We showed that while Aβ transgenic worms have increased susceptibility to the effects of PTZ, PLD functional ablation confers a protective effect in PTZ-susceptibility induced by Aβ expression (Fig. 4I) (Supplementary Table S5). Taken together, these observations indicate that PLD ablation protects from Aβ-induced deficits.

## Discussion

PLD is a lipid modulatory enzyme involved in multiple aspects of cell physiology, such as signaling and membrane trafficking processes. Furthermore, it has been implicated in pathologic conditions like cancer and neurodegenerative diseases^8,25^. To understand the role of PLD, mutant models have been developed using different organisms, such as mice^5,15^ and flies^13,14^. While in mammals there are two canonical isoenzymes, PLD1 and PLD2, in flies and nematodes there is only one PLD ortholog^16^.

Here, we present an extensive characterization of the effects of PLD ablation using a *C*. *elegans* model. We show that PLD mutants, *pld-1*, have decreased levels of PA (Fig. 1A) and that PLD is the only source of PEtOH (a specific product of PLD activity) (Fig. 1C). This is in accordance with the results from another model organism, showing that with the genetic ablation of PLD in drosophila, PLD was the only source of PLD activity^13^. In mammals, the contribution to total PLD activity by either PLD1 or PLD2 depends on the tissue or cell type. For instance, it was previously shown in the brain that while PLD2 ablation leads to decreased total PLD activity^5^, no changes are observed in total PA levels^5,26^. In another study, it was observed that PLD1 ablation led to decreased PLD activity in the liver and while there were no differences in total PA levels, PA supplementation restored autophagy deficits in PLD1 knock-out cells^27^. Moreover, we show that PLD metabolizes ethanol, producing PEtOH, and that *pld-1* animals are more sensitive to ethanol-induced slowing (Fig. 1F). While PEtOH measurements provide direct evidence that PLD metabolizes ethanol acutely, we observed no differences in total ethanol levels after one hour treatment, suggesting, at least in *C*. *elegans*, PLD has a minor role for ethanol elimination. The increased sensitivity of *pld-1* animals to acute ethanol effects could be explained alternatively by a diversion of PA synthesis or due to potential protective effects by PEtOH. Accordingly, previous reports showed that, not only chronic ethanol exposure, with associated production of PEtOH^28,29^, but also PEtOH itself^30^, induce resistance to acute ethanol effects on membranes, which suggests that PEtOH could have a protective role in acute ethanol exposure. Since in mammals there are two PLDs, the specific role of either PLD1 or PLD2 in ethanol-induced toxicity should be differentially studied.

Our results show that PLD is not essential for the survival or for the normal functioning of various behavioral tasks in a *C*. *elegans* model (Fig. 2). In mice, both PLD1 and PLD2 knock-out animals are viable^5,15^. Even though it was reported that PLD1 or PLD2 ablation led to decreased juvenile brain volume and to social and object recognition deficits^31^, no major behavioral deficits were observed by other research groups in PLD2 knock-out adult mice^8,26^, apart from olfaction deficits in aged animals^26^.

Concerning biometric and metabolic parameters, deletion of either of the PLD enzymes led to elevated body weight and increased adipose tissue content in aged animals^32^. However, while others did not observe elevated body weight in PLD2 knock-out animals^33^, PLD1 knock-out animals presented not only increased hepatic weight, but also increased triacylglycerol levels and increased cholesterol levels^27^. Here, in a *C*. *elegans* model, we observe that *pld-1* animals present both increased size and lipid stores (Fig. 3). We show that PLD is a main source of PA in *C*. *elegans* (Fig. 1A), so it is possible that the effects of PLD perturbation could be due to altered PA metabolism. Therefore, other enzymes, which modulate PA levels, such as lipin (*i*.*e*., a phosphatic acid phosphatase), could potentially be involved in the regulation of convergent physiologic mechanisms. Curiously, it was previously shown that silencing of the *C*. *elegans* homolog of lipin (*lpin-1*) leads to reduced body size and defects in lipid storage^34^. It was also observed in *C*. *elegans* that depletion of PC synthesis enzymes stimulates sterol regulatory element binding protein (SREBP) transcription factors, increases both fat-7 levels and lipid stores^35^ and in a follow-up study lpin-1/Lipin-1 knock-down reduced the effects of PC-depleted conditions in a PA dependent way^36^. Since lipin-1 converts PA to DAG, our data raises the possibility that in *C*. *elegans*, the PA species derived from PLD, could be involved in the mechanism of lipid stores regulation observed upon *lpin-1* knock-down. Moreover, lipid stores have been previously shown to be consumed through a specialized form of macroautophagy called macrolipophagy^37^. Since, previous reports have shown a role for PLD1 in macroautophagy^38^ and in the regulation of lipid stores^27^ we hypothesize that this could be an alternate explanation for this phenotype.

To understand the impact of PLD ablation in a neurodegenerative disease *C*. *elegans* model, we crossed *pld-1* animals with the CL2355 strain, which overexpresses human Aβ in neurons. This Aβ strain was shown to present multiple behavioral deficits and decreased survival^18–20^. We had previously observed that *C*. *elegans* expressing Aβ presented decreased worm size, and remarkably, *pld-1*; Aβ animals had a significant recovery in animal volume (Fig. 4B), with a major impact in worm survival (Fig. 4C). Additionally, PLD ablation in *pld-1*; Aβ animals had a protective effect in motor behaviors (Fig. 4F and G) and in the defective responses to serotonin (Fig. 4H) and PTZ (Fig. 4I). This is in line with previous results showing that Aβ leads to increased total PLD activity and that PLD2 genetic ablation ameliorates synaptic and behavioral deficits in an amyloidogenesis AD mouse model, independently of an effect on APP processing^5^. Interestingly, in an unbiased gene expression study, it was observed in another *C*. *elegans* strain expressing human Aβ that the toxic peptide leads to gene expression changes that overlap with changes induced by the membrane pore-inducing toxin, Cry5B^39^, suggesting that membrane damage mechanisms could be important pathways induced by Aβ. In fact, PLD has been shown to be involved in membrane damage pathways^40^. Moreover, fly PLD was shown to be required to support rhabdomere volume during illumination, a task that relies on active membrane turnover^13^. Previously it had been shown that PLD overexpression also has a deleterious impact, leading to degeneration of rhabdomeres^14,16^. These reports in drosophila, show that either a decrease or increase in PLD levels can have a functional impact in fly rhabdomeres, which highlights the importance of tightly regulating PLD activity in physiologic processes with high membrane turnover.

To our knowledge, this is the first report evaluating the impact of PLD ablation in *C*. *elegans*. We further present observations that support PLD as a downstream pathway of Aβ’s interaction with membranes or a putative membrane receptor. Overall, we observe multiple phenotypes that somewhat phenocopy previous observations in other PLD genetic models. Future studies will be able to benefit from *C*. *elegans* PLD models as relevant tools to study the role of PLD in physiologic and pathologic mechanisms.

## Methods

### Nematode Strains and culture conditions

Strains used in this work were acquired from the *Caenorhabditis* Genetics Center, namely Bristol N2; RB1737, *pld-1*(*ok2222*) II; CB1370, *daf-2*(*e1370*) III; CF1038, *daf-16*(*mu86*) I; PS2627, *dgk-1* (*sy428*) X; PR811, *osm-6* (*p811*) V; PR696, *che-1* (*p696*) I, and CL2355, *smg-1*(*cc546*) *dvIs50* I, *dvIs50* [pCL45 (*snb-1*: Abeta 1–42::3’ UTR(long) + mtl-2::GFP]. The CL2355 strain has a pan-neuronal expression of Aβ_1–42_, which is inducible by temperature up-shift to 23 °C. The transgenic strain is referred to as neuronal Aβ strain. All the strains were backcrossed to Bristol strain N2 eight times. Strain CL2355 was crossed with strain RB1737 using standard procedures, generating *pld-1;* Aβ animals. Worms were grown in agar plates with nematode growth media (NGM) at 20 °C as previously described^41^. In the experiments where the Aβ strain was used, the animals grew 36 h at 16 °C followed by temperature upshift to 23 °C. Synchronized cultures were used for all assays and obtained through egg laying, by collecting embryos laid by adult animals during 3 h or using a bleaching procedure, by treating animals with an alkaline hypochlorite solution (0.5 M NaOH, 2.6% NaClO) for 7 min^42^.

### Lipid analysis

N2 and *pld-1* animals were incubated in the presence or absence of ethanol (1%) for 1 h and all samples were immediately collected, frozen in liquid nitrogen and stored at −80 °C until further processing. Approximately 100 animals were used per sample. Lipids were subsequently extracted by a chloroform/methanol extraction, as previously described^43,44^. Lipid species were analyzed using a 6490 Triple Quadrupole LC/MS system (Agilent Technologies, Santa Clara, CA) operated in multiple reactions mode (MRM). PA and PEtOH levels were quantified by comparing to spiked internal standards diC17-PA and diC16-PEtOH (Avanti Polar Lipids). Lipid concentration was normalized by molar concentration across all species for each sample, and the final data is presented as the mean mol %^43,44^.

### Ethanol susceptibility assay

Plates (60 mm) with 8,5 mL NGM and 1 mL of ethanol at final concentrations ranging from 100 to 500 mM (adjusted to the volume of the agar) were freshly prepared. Plates were then sealed for 2 h at room temperature (RT) and copper rings (10 mm diameter) were melted onto the agar surface. Day three synchronized worms were placed in plates in the absence of food for 30 min prior to the assay, and then transferred to the ethanol plates. After 20 min of exposure, one-min videos were taken with an Olympus PD72 digital camera attached to an Olympus SZX16 stereomicroscope. Mean worm speed was quantified using the dVision software (Delta Informatika ZRt, Budapest, Hungary)^45^.

### Ethanol Assay Kit

Worms were synchronized by egg laying and grown at 20 °C until they reached the adult stage (day three post-hatching). Ethanol levels were assayed using the ethanol assay kit (MAK076, Sigma). Briefly, nematodes were washed in M9 buffer and 50 worms were placed per well to a final volume of 50 µL with the ethanol assay buffer. The master reaction mix was prepared according to the specifications of kit. To each well, 50 µL of the master reaction mix were added, and incubated for 60 min at room temperature, after which the absorbance at 570 nm (A_570_) was measured. The concentration of ethanol was determined based on the A570 of standards provided in the kit.

### Fecundity and Egg Viability Assay

Animals were synchronized by egg-laying. At day three, animals were individually transferred into 30 mm plates (10 animals per strain) with a bacterial lawn of 10 mm of diameter. After 5 h, all worms were removed and the total number of eggs in each plate was counted. The plates were maintained at 4 °C in order to delay egg hatching, while counting them. Egg viability was determined as the percentage of eggs that were able to hatch over the following 24 h.

### Brood Size

Brood size evaluation was performed as previously described^46^. Briefly, 15 L4 animals, per strain, were kept at 23 °C in individual plates (30 mm) with a bacterial lawn of 10 mm of diameter and allowed to lay eggs. Animals were transferred to fresh plates daily and total progeny counted every day for 8 days.

### Development

Synchronized worms through hypochlorite treatment were placed in a freshly seeded NGM plate (50 animals per strain). After 48 h, the percentage of animals which were in the L4 to adult stage was scored.

### Motility assay

The motility assay was performed as previously described^47^ at RT (~20 °C), using day three synchronized animals grown at 20 °C. Five animals were placed simultaneously in the middle of a freshly seeded plate, equilibrated at 20 °C. Animals remaining inside a 10 mm circle after 1 min were scored as locomotion-defective. At least 150 animals were scored for each strain in three independent assays.

### Chemotaxis assay

Chemotaxis assays were performed based on the assays previously developed^48,49^. Well-fed, synchronized adult day three animals (through bleaching) were collected and washed with S-Basal buffer three times to remove all the food. The assay plates (20 g/L agar-agar; 5 mM KH_2_PO_4_; 1 mM CaCl_2_; 1 mM MgSO_4_) were prepared by adding 1 µL of 5 M NaCl or 0.1 M isoamyl alcohol (IAA) 10 mm from the center of the plate on one side. On the opposite side of the plate, a 1 µL drop of water or of 100% ethanol was added. Afterwards, 1 µL of 1 M sodium azide was additionally added to the preexisting spots to paralyze the animals. Worms (~100–200) were quickly transferred to the center of the plate and the excess of liquid removed with a filter paper. The assay plates were incubated at 20 °C for 60 min and the chemotaxis index was scored as the (number of animals at attractant - number of animals at counter-attractant)/Total number of animals in assay. Three to four independent assays were conducted with at 100 to 150 animals per assay per plate. For each strain, two to three plates were tested per assay.

### Short-term and Long-term Associative Memory Assays

A *C*. *elegans* odorant preference assay protocol was adapted from previous reports^50^. A chemotaxis assay using 1 M diacetyl (Sigma-Aldrich) was performed as described above in order to confirm that the animals’ genotype did not affect the chemotaxis index to diacetyl (naive group). To assess the 1× associative learning, well-fed day three animals were starved for 1 h in the presence of diacetyl and placed on the lid of the plates. Right after the starvation period, the chemotaxis index was scored again to assess learning (represented as starv 1 h in the graph). After conditioning the worms with diacetyl, worms were placed on NGM plates with food for 1 h. To test for short-term associative memory of the food-diacetyl association, the chemotaxis index was again evaluated (short memory). The long term associative memory was performed 24 h later (long memory). As a control, the same conditions were tested in fed animals to test for habituation.

### Lifespan

Synchronized adult animals were placed on 60 mm NGM plates at 20 °C, examined every day and scored as dead if no mechanical response was obtained after gentle touch with a platinum wire. Animals were transferred to fresh plates every 2 days to avoid starvation and progeny contamination. Animals were censored from the analysis if lost, desiccated on the edge of plates, if showing extruded gonad or suffered internal progeny hatching. Evaluations ended after all animals were dead or censored. The lifespan evaluation at 23 °C was performed as described above. Experiments were performed blindly.

### Dopamine susceptibility assay

Worms were synchronized by egg laying and grown at 20 °C until they reached the adult stage (day three post-hatching). Different solutions of dopamine hydrochloride (Sigma-Aldrich) (concentrations ranging from 100 to 800 mM) were prepared and 1 mL of the solution was added to NGM plates (60 mm) without OP50 and allowed to dry for 60 min. The adult worms were placed in each plate (10 animals per strain) and after 30 min their motor phenotype was assessed according to the following scores: 0 for normal locomotion, 1 for sluggish/slower movement, 2 for semi-paralysis (body bends without moving), 3 for paralysis (only the head moves after mechanical stimulation) and 4 for death^21^.

### Biometric analysis

Biometric analysis was performed at 72 h after egg laying. Length and diameter measurements were calculated using ImageJ software®, and volume was determined by treating worms as cylinders (v = π*r^2^*l)^51^.Biometric analysis of F2 animals was performed using the progeny of synchronized worms grown in NGM plates with or without cholesterol. Pictures were acquired 72 h after hatching. Worms were photographed using an Olympus PD72 digital camera attached to an Olympus SZX16 stereomicroscope.

### Nile Red Staining

The Nile red staining protocol was adapted from previously described protocols^52^. Nile red (Molecular Probes) was dissolved in a 0.5 mg/mL acetone stock solution. On the day of the assay, the stock solution was freshly diluted in 1× PBS to a final concentration of 1 µg/mL. Egg laying synchronized worms were washed 3 times with M9 and transferred to a conical tube containing Nile Red. Worms were incubated at 20 °C for 2 h and washed 3 times to remove the excess of dye before imaging^52^.

### Confocal Imaging

For confocal dynamic imaging and quantification of Nile Red staining, live animals were paralyzed with 3 mM levamisole (Sigma-Aldrich) and mounted on a 3% agarose pad. All images were acquired on an Olympus FV1000 (Japan) confocal microscope, under a 60× oil objective and resolution of 640 × 640. A z-series image was acquired for all treated worms using a 594 nm laser. The pinhole was adjusted to 1.0 Airy unit. The images were analyzed and processed using ImageJ software®.

### Thrashing analysis

Single synchronized adult animals were transferred to a 10 µL drop of M9 buffer. After 1 min animals were filmed at a rate of 15 frames-per-second, in a total of 600 frames, using an Olympus PD72 digital camera attached to an Olympus SZX16 stereomicroscope. The number of total body bends per 30 seconds was then quantified using ImageJ software® with the wrMTrck plugin^53^.

### Defecation Motor Program (DMP)

Adult well-fed synchronized worms (10 per strain) were placed on NGM plates (90 mm), freshly seeded with OP50, for 10 min. Each animal was individually evaluated for exactly 10 min and the total number of DPMs was counted in this interval. The results were expressed as the average time (in seconds) between each successive cycle.

### Pharyngeal Pumping

Synchronized day three worms were placed on plates (90 mm) freshly seeded with OP50. After 1 h, worms present in the border of the OP50 were selected and recorded with an Olympus PD72 digital camera attached to an Olympus SZX16 stereomicroscope (10 worms per strain). Each movie was recorded for 30s and the total number of pharyngeal contractions was counted in this interval.

### Serotonin Sensitivity Assay

The serotonin assay was performed as previously described^18^. Serotonin (creatine sulfate salt, Sigma-Aldrich) was dissolved in M9 buffer to 1 mM. Synchronized three-day worms underwent temperature upshift to 23 °C to activate transgene expression of the Aβ strain, prior to the assay. Worms were washed with M9 buffer and placed in 200 µL of serotonin 1 mM in a 96-well plate. The animals were scored as active or paralyzed in each well after 5 min.

### PTZ susceptibility Assay

The PTZ susceptibility assay was adapted from others^24^. Plates (30 mm) were prepared with 3 mL NGM each (without food). A stock solution of 100 mg/mL PTZ (Sigma-Aldrich) and the respective dilutions (20, 40, 50, and 80 mg/mL) were prepared. To each plate, 250 µL of PTZ was added. The plates were allowed to dry for 90 min in a flow chamber. Afterwards, 5 times concentrated OP50 was added to the center of each plate. The susceptibility assay was evaluated by placing worms in the bacterial lawn in each plate. After 30 min, each worm’s phenotype was evaluated using the following score: 0 for no major decrease in worm movement, 1 for sluggish/slower movement, 2 for semi-paralysis (body bends without moving), 3 for paralysis (only the head moves after mechanical stimulation) and 4 for death.

### Statistical analysis

A confidence interval of 95% was assumed for all statistical tests. Normality was tested using the Kolmogorov-Smirnov test, and was assumed for all tested variables. In all experiments comparing two variables, the data was analyzed with Student’s t-test with the Levene’s test for equality of variances. When more than two variables were analyzed, a one-way analysis of variance with the Levene’s test for equality of variances and a post-hoc Tukey test for multiple comparisons was performed. The ethanol and dopamine susceptibility assays were analyzed using a two-way analysis of variance with the Levene’s test for equality of variances and a post-hoc Tukey test for multiple comparisons. The PTZ susceptibility assay was analyzed using a repeated measures analysis of variance with the Mauchly’s test for sphericity and a post-hoc Games-Howell test for multiple comparisons. Lifespan was evaluated by the log-rank (Mantel-Cox) test and the Hazard Ratio obtained from a Cox regression model, using the strain as a categorical covariate and a simple contrasts analysis. Statistical analysis was performed using GraphPad Prism 6.01 software® and SPSS 22.0 (SPSS Inc.)

## Electronic supplementary material

Supplementary Data
