# Supplementary material for: Phospholipase D functional ablation has a protective effect in an Alzheimer’s disease Caenorhabditis elegans model
Source: Sci Rep. 2018 Feb 23;8:3540. doi: 10.1038/s41598-018-21918-5 (PMC5824944; doi:10.1038/s41598-018-21918-5)
Supplement: Supplementary file 1 — Supplementary Data [file 41598_2018_21918_MOESM1_ESM.doc]

**Phospholipase D functional ablation has a protective effect in an Alzheimer’s disease *Caenorhabditis elegans* model**

Francisca Vaz Bravo, Jorge Da Silva, Robin Barry Chan, Gilbert Di Paolo, Andreia Teixeira-Castro, Tiago Gil Oliveira

**Supplementary Data**


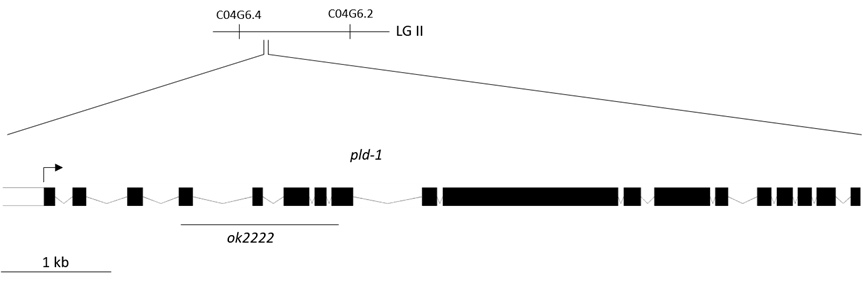


**Supplementary Figure S1: *pld-1* encodes the *C. elegans* ortholog of phospholipase D.** Genetic map of the *pld-1* region of LG II. Intron–exon structure of *pld-1* (RB1737), inferred from cDNA sequences. Boxes, Coding regions; lines, untranslated regions, arrow, direction of transcription. The *pld-1* open reading frame is 4285 bp within a 4659 bp cDNA. The extent of the 1430 bp deletion is depicted.

**
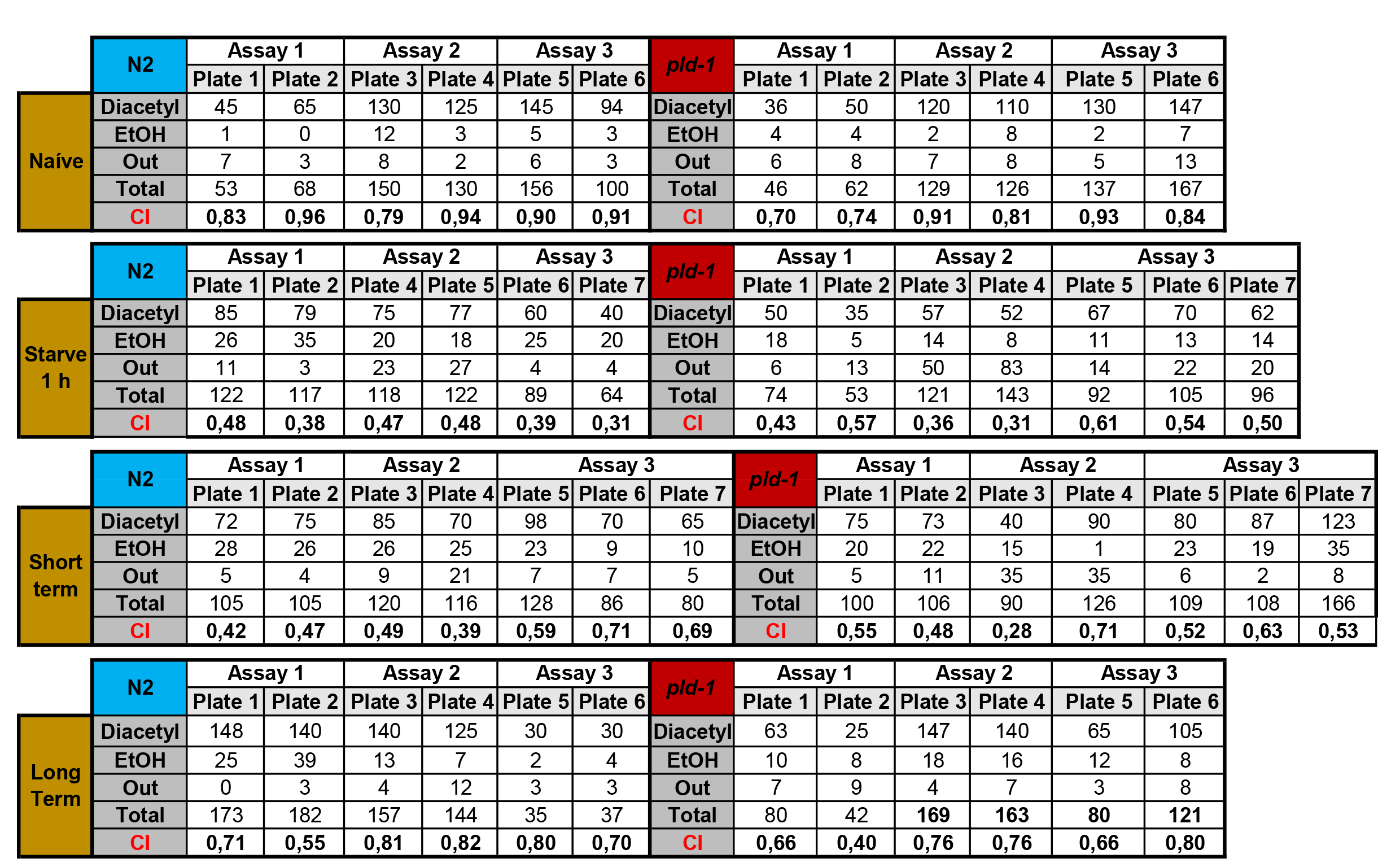
**

**Supplementary Table S1.** **Raw data from learning and memory assay represented in Fig. 2F.** The chemotaxis index was scored to assess the naíve, learning (starve 1 h), short term and long term memory Three independent experiments were performed and the number of animals used in each assay are represented in the table.


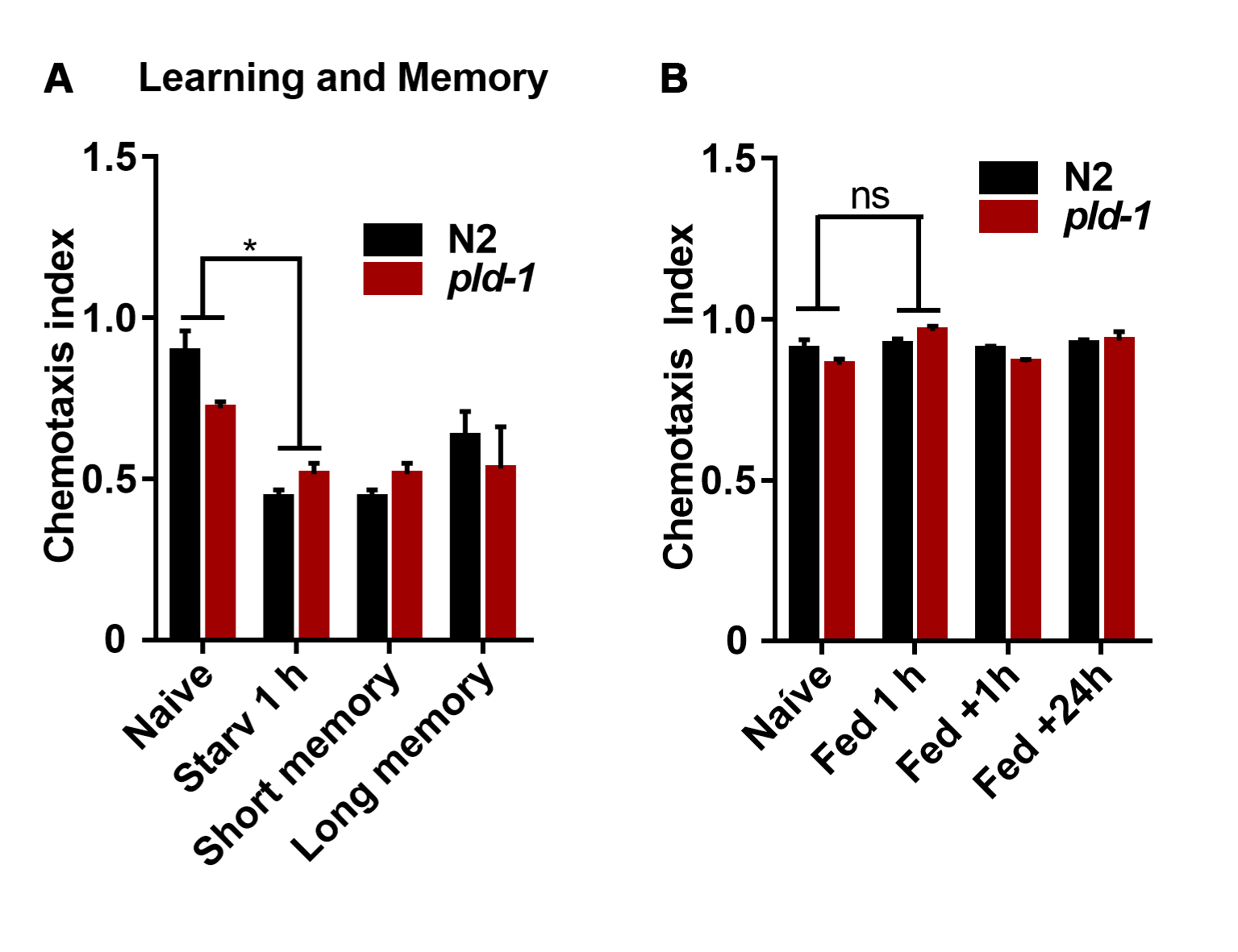


**Supplementary Figure S2. (A) Additional representative experiment of learning and memory independent assay**  ***pld-1* animals have no deficit in an associative learning task.** N2 and *pld-1* short-term and long-term associative memory profile after 1 h and 24 h of conditioning. Three independent experiments were performed (n = 200 worms per assay). **(B) *pld-1* animals in fed conditions and in the presence of diacetyl have a normal chemotaxis upon repetitive exposure.** N2 and *pld-1* chemotaxis index profile after 1 h and 24 h the fed period. Representative figure of two independent experiments performed (3 chemotaxis assay plates per strain, n=100 worms per chemotaxis assay plate) experiment).

**
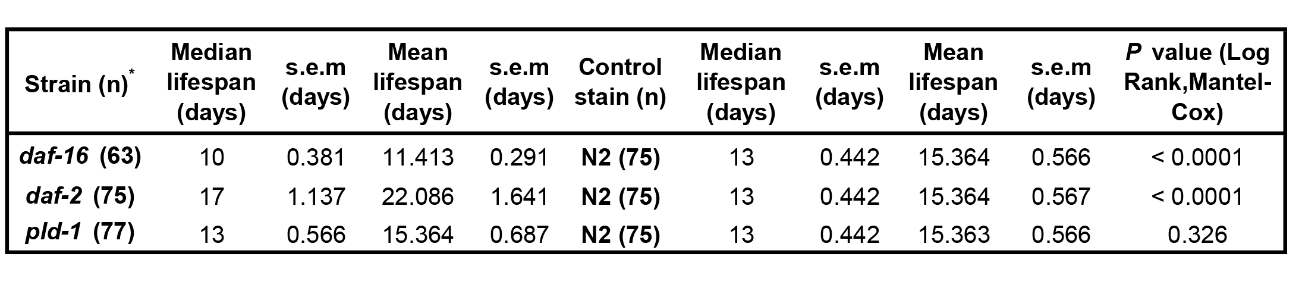
**

**Supplementary Table S2.** ***pld-1* animals have a normal lifespan.** Survival statistics analysis of N2, *pld-1*, *daf-16* and *daf-2* animals at 20ºC.


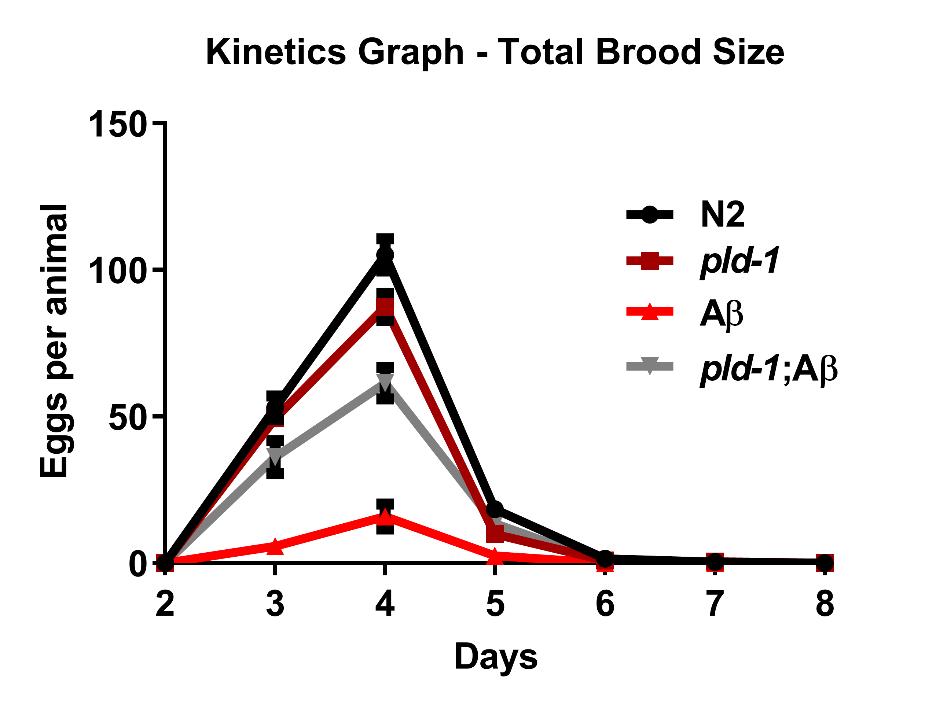


**Supplementary Figure S3. PLD function ablation increases the number of progeny in Aβ animals.** Number of eggs layed in adult hermaphrodite N2, *pld-1,* Aβ and *pld-1*; Aβ worms was counted for 8 days. The progeny of 15 worms per strain was evaluated per day. Representative figure of two independent experiments performed.


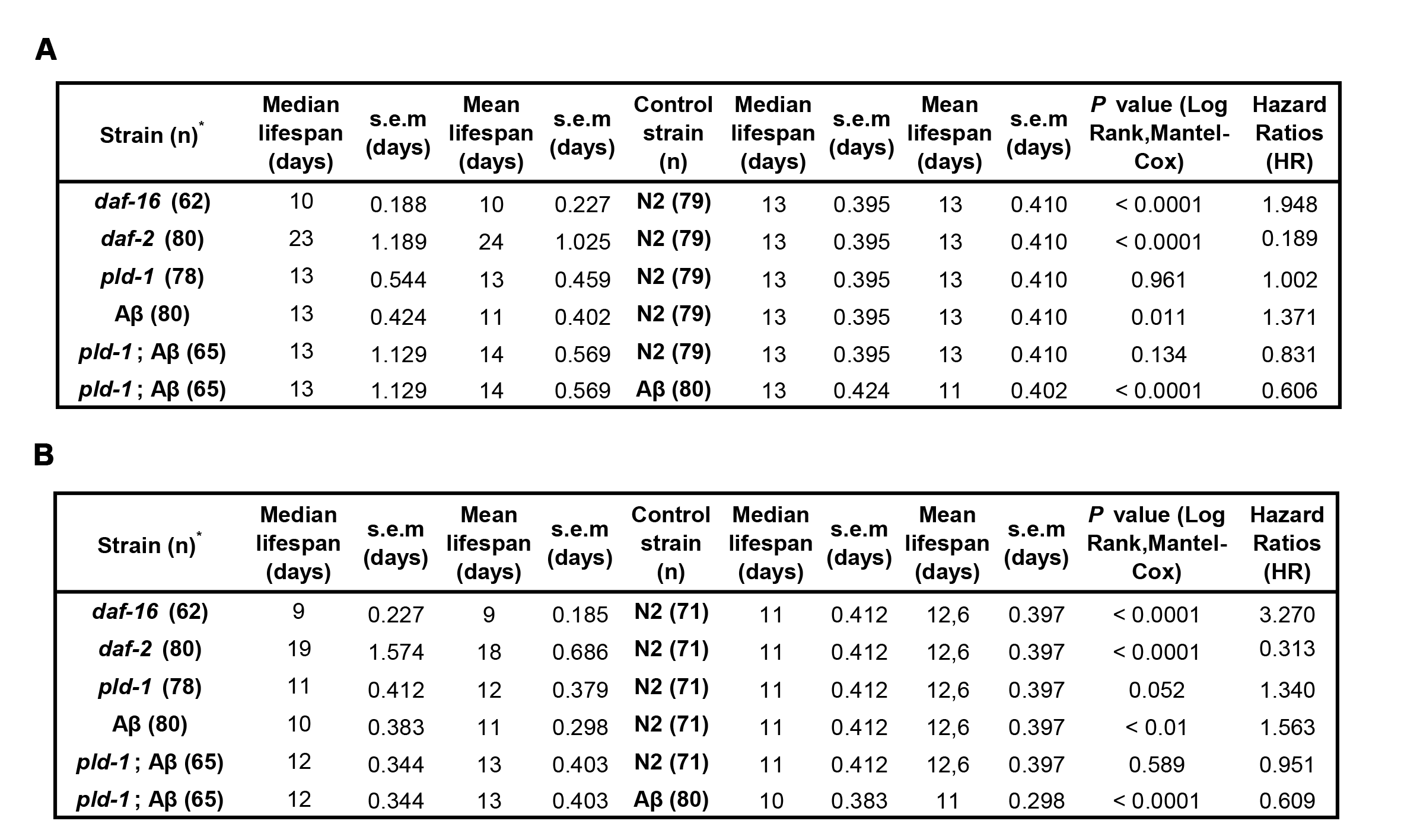


**Supplementary Table S3. PLD ablation restores survival of Aβ transgenic animals. (A)** Survival statistics analysis of experiment #1. **(B)** Survival statistic analysis of experiment #2 of N2, *pld-1*, Aβ, *pld-1;* Aβ, *daf-16* and *daf-2* animals at 23ºC.

**
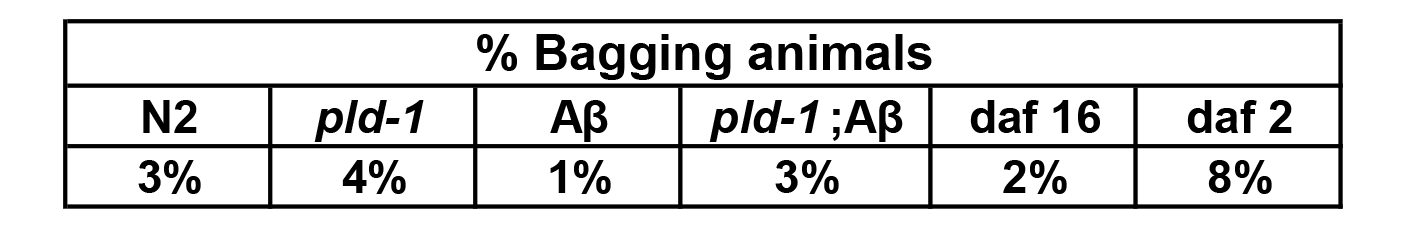
**

**Supplementary Table S4.** Percentage of bagging animals present in survival experiment of N2, *pld-1*, Aβ, *pld-1;* Aβ, *daf-16* and *daf-2* animals at 23ºC.


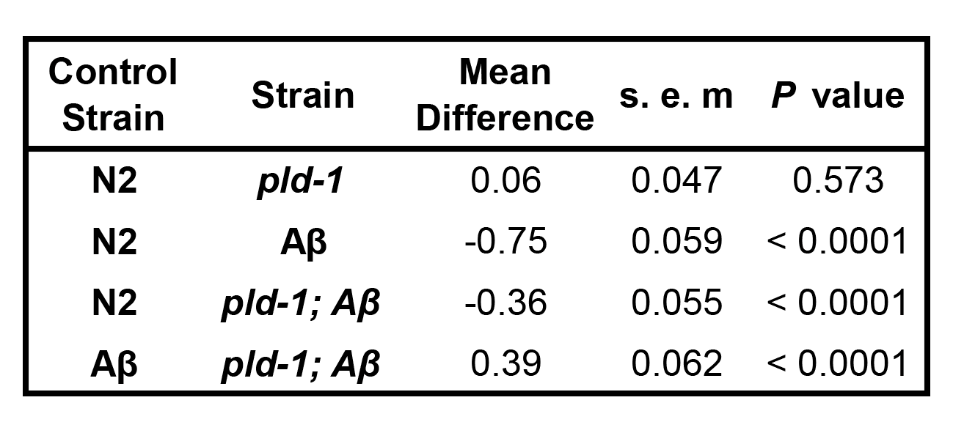


**Supplementary Table S5:** Repeated measures analysis of variance of the PTZ susceptible assay in *C. elegans.* F (3, 3.335) = 3309.545, p < 0.0001.
